# From collective efficacy and negative emotions toward management and conservation of wetlands: the mediating role of social identity

**DOI:** 10.3389/fpsyg.2025.1362750

**Published:** 2025-03-14

**Authors:** Naser Valizadeh, Vahid Karimi, Khadijeh Bazrafkan, Hossein Azadi, Hassan Azarm

**Affiliations:** ^1^Department of Agricultural Extension and Education, School of Agriculture, Shiraz University, Shiraz, Iran; ^2^Department of Agricultural Extension and Education, Faculty of Agriculture, Tarbiat Modares University (TMU), Tehran, Iran; ^3^Department of Socio-Economic and Agricultural Extension Research, Agricultural Research, Education & Extension Organization (AREEO), Tehran, Iran; ^4^Department of Economics and Rural Development, Gembloux Agro-Bio Tech, University of Liège, Gembloux, Belgium; ^5^Department of Agricultural Economics, School of Agriculture, Shiraz University, Shiraz, Iran

**Keywords:** wetland communities, we thinking system, collective pro-environmental behaviors, destruction of wetlands, farmer identity

## Abstract

Wetlands are among the most valuable natural resources on Earth. However, many have been destroyed in recent decades. One suggested solution for their sustainable use is the creation of collective management and protection strategies. These measures would involve stakeholders at various levels. Therefore, in this study, the encapsulation model of social identity in collective action is used to increase understanding of how participation in the collective management and protection of wetlands is strengthened. The aim of this study was to examine farmers’ willingness to engage in collective protection and management activities for Helleh Wetland. To achieve this, a cross-sectional survey was conducted among farmers living near the Helleh Wetland in Iran. The results indicated that the encapsulation model of social identity in collective action effectively explained the farmers’ intentions to participate in the collective management and protection of the wetland. According to the results, social identity and negative emotions had significant relationships with the intention toward participation in collective management and protection. Similarly, collective efficacy was also found to be related to the intention toward participation in collective management and protection. The results also indicated that using the encapsulation model of social identity in collective action to motivate farmers to participate in wetland management and protection can be effective. However, this approach will be most successful if environmental management authorities prioritize addressing and removing past negative experiences related to participation. This study offers insights into the socio-psychological factors that influence intentions to participate in collective wetland management and conservation. The findings also provide valuable implications for managers, policymakers, and decision-makers, helping them to effectively encourage participation in collective wetland management and conservation.

## 1 Introduction

Wetlands are one of the most dynamic ecosystems on Earth that have direct impacts on environmental sustainability and human survival (Woodward and Wui, 2001; Ramsar Convention on Wetlands, 2018; Xue et al., 2018). Wetlands are defined as areas of land that are covered by swamps, vegetation, marshes, or water, which can be permanent or temporary, static or flowing, and may be either fresh or saline. Additionally, in some cases, areas of seawater with a tidal depth not exceeding 6 meters are also classified as wetlands (Ibrahim et al., 2012; Hu et al., 2017). Wetlands provide a livelihood for many people around the world, particularly in developing countries like Iran, Pakistan, and Egypt. They are also highly valued in various traditional and local cultures (Sinthumule, 2021; Aryal et al., 2021). Wetlands serve a variety of functions across environmental, economic, and socio-cultural dimensions. Key environmental functions include water storage, wildlife and biodiversity conservation, storm and flood protection, coastline stability, erosion control, prevention of saline intrusion, groundwater recharge, and maintaining local climate stability (Zebardast et al., 2021; Costanza et al., 2021). The economic functions of wetlands include protecting agricultural reserves, producing timber, supplying energy, providing fodder and pasture for livestock, offering crops and medicinal products, creating tourism opportunities, and supporting local employment through activities like fishing and related industries (Dechasa et al., 2021; Valizadeh et al., 2021). In addition, wetlands play a crucial role in the socio-cultural dimension. For instance, they help maintain the cultural traditions and customs of local communities. Wetlands also support the lifestyle of nearby households and help prevent migration. These socio-cultural functions are essential for the preservation of wetlands (Ostad-Ali-Askari, 2022). These functions significantly increase the importance of preserving wetlands for both present and future generations, particularly in developing countries like Iran (Qiu and Fan, 2016; Everard, 2017; Davidson, 2018; Bhowmik, 2022).

Despite their vital and valuable functions, wetlands have faced significant environmental challenges in recent years. Climate change, depletion of groundwater resources, and human activities like agriculture have become the main threats to wetlands over the past decade. These issues have had particularly destructive effects on wetlands, especially in arid and semi-arid regions (Karimi et al., 2018; Moomaw et al., 2018; Finlayson et al., 2019; Bhowmik, 2022). Studies (Junk et al., 2013; Davidson, 2014) show that the area of wetlands has sharply declined in the twentieth century. For instance, Iran, which has 141 wetlands and 1.4% of its land area covered by wetlands (Iran Environment Organization, 2019), is one of the countries that has faced numerous challenges in sustainable wetland management and use over the past decade. The unsustainability of wetlands in Iran results from several interconnected issues, including mismanagement, severe droughts, development without proper evaluation, excessive agricultural expansion, illegal well drilling, and land use changes in wetland areas (Madani, 2021; Faghani et al., 2023). Despite the importance of each of these factors in the destruction of wetlands in Iran, the lack of collective or participatory actions is one of the key reasons for the unsustainability of wetland management programs. In other words, farmers and local communities living around the wetlands are often not involved in many management and protection efforts related to wetlands (Rees et al., 2015; Van Zomeren et al., 2008; Iyer et al., 2007).

In a collective approach to wetland protection and management, often-overlooked stakeholders, such as farmers and local communities, have the chance to take crucial steps in protecting and rehabilitating wetlands. However, when such an approach is not used, it often results in the exclusion of key stakeholders. This neglect can even lead to opposition from these groups toward wetland protection efforts. Farmers’ participation in collective measures for wetland management and protection can foster the co-production of knowledge (Zarei et al., 2020; Bamberg et al., 2015) and offer effective solutions for the environmental, social, and economic adaptation of wetlands. Unfortunately, beliefs and perceptions regarding the effectiveness of collective action in addressing issues like wetland degradation have not yet been institutionalized among Iranian environmental policymakers, decision-makers, and program planners (Faghani et al., 2023; Valizadeh et al., 2022b). As a result, efforts to increase farmers’ participation in collective actions for wetland management and protection are rarely made. Focusing on farmers’ intentions toward collective and participatory actions can lead to a better understanding of the need to preserve wetlands. It can also promote social learning in response to environmental crises, increase motivation for participation, and enhance the effectiveness of collective actions (Keshavarz and Karami, 2016; Rees and Bamberg, 2014). Involving local stakeholders, such as farmers, in management programs and policies can help identify and develop adaptive and efficient agricultural practices within communities living around wetlands (Karimi et al., 2020). Therefore, active collective action and effective communication among farmers, local professionals, managers, and policymakers are essential for creating opportunities to enhance and sustain wetlands in Iran in the future (Yazdanpanah et al., 2015; Bamberg et al., 2015). However, this requires identifying the factors that influence farmers’ participation in collective actions for the protection and management of wetlands. It also emphasizes the need for communication systems between socio-psychological factors as a key aspect of any wetland management program.

The research on the factors influencing the participation of farmers and local communities in wetland management and protection, both globally and in Iran, highlights the importance of economic, demographic, and political factors. For example, Ghanian et al. (2022) used an economic approach based on contingent valuation to examine the factors affecting stakeholder participation in the protection of Iran’s wetlands. Their findings indicated that education, employment status, living expenses, and marital status were significantly related to stakeholders’ participation. Notably, their study also revealed that living expenses and marital status were negatively associated with participation in wetland protection activities. In this study, participation was defined as involvement in the design, implementation, and evaluation of wetland management programs. Zhang et al. (2011) explored the factors influencing farmers’ willingness to convert cultivated land into wetlands in China. Their study identified key demographic factors—such as age, education, cultivated area, geographical location, and the perceived risks and benefits—as the most important determinants of farmers’ willingness to participate in land-to-wetland conversion. Rutter et al. (2022) conducted one of the few studies focusing on psychological variables, such as ecological awareness, connection to wetlands, and wildlife recreation, in relation to participation in wetland conservation. Their research found that these three factors were positively and significantly associated with respondents’ willingness to participate in wetland protection effort.

Most previous research has focused on the relationship between economic, demographic, and political factors and farmers’ participation in wetland management and protection. However, few studies have explored the socio-psychological factors influencing farmers’ participation in these efforts. This research aims to fill that gap. The study is innovative in several ways. First, it examines wetland protection and management from a collective action perspective. In other words, this study seeks to institutionalize the understanding among stakeholders involved in wetland protection and management that major environmental crises, such as wetland unsustainability, are multi-stakeholder issues. Therefore, any solution for protection and management must be based on “collective will” rather than “individual will.” Second, to the best of the authors’ knowledge, no study has explored farmers’ intentions to participate in collective wetland protection and management actions in either Iran or globally. Therefore, this study can serve as a turning point for decision-makers, managers, academics, and practitioners involved in wetland management and protection, offering pragmatic insights that can be applied to sustain wetlands. Third, this study introduces the concept of “we-thinking/social identity” as the foundation of any collective action in wetland management. It suggests that frameworks and theories based on “individual action” should not be solely relied upon to encourage farmers’ participation in collective efforts. In other words, for farmers to effectively engage in collective action, interventions should be based on social identity models of collective action. The aim of this study is to analyze farmers’ willingness to participate in the collective protection and management activities of Helleh Wetland. In doing so, the study seeks to answer the following questions:

1. What are the main determinants of willingness to participate in collective protection and management activities of the wetlands?
2. Can the encapsulation model of social identity in collective action (EMSICA) help to increase the understanding of willingness to participate in the protection and management of wetlands?

## 2 Theoretical framework

**Figure 1:**
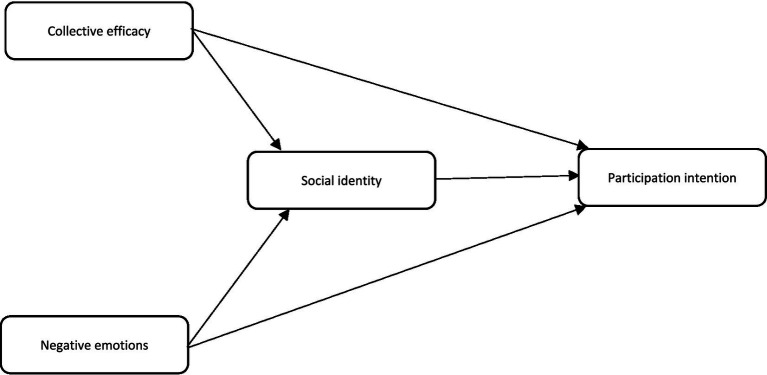
The encapsulation model of social identity in collective action.

Research on collective actions typically focuses on the factors and motivations behind group-based activities, which often arise from shared emotions within a group (Gulliver et al., 2020). The literature on collective action suggests that when individuals face significant challenges, such as wetland destruction or the depletion of natural resources, they assess these issues in terms of the interests of the group to which they belong. These emotions, along with a sense of responsibility toward the group, can lead to feelings of dissatisfaction and anger regarding the current situation, thereby motivating individuals to engage in collective action (Thomas et al., 2009; Van Zomeren et al., 2004). This theory, known as the *Social Identity Model of Collective Action (SIMCA)*, was first proposed by van Zomeren et al. (2008). Thomas et al. (2009) developed the SIMCA theory through an integrated meta-analysis of global studies on collective action. Their findings highlighted the complementary role of social identity in collective action. Building on this, they introduced a new theory called EMSICA. EMSICA is based on four variables: intention toward collective action (the outcome variable), and social identity, collective efficacy, and negative emotions (the predictors) (Bamberg et al., 2015; Valizadeh et al., 2022b). Based on the theoretical literature discussed, EMSICA was conceptualized as shown in Figure 1. From this framework, five research hypotheses were developed. Below, the relationship between each of the predictor variables (collective efficacy, social identity, and negative emotions) and the response variable (intention) is examined in more detail.

### 2.1 The relationship between collective efficacy and intention

Collective efficacy refers to individuals’ belief in the effectiveness of participating in collective action. In other words, it is the perception within a group (such as farmers) that they can successfully collaborate to achieve important goals (Faghani et al., 2023). For instance, when farmers believe that their collective efforts can prevent the destruction of wetlands and protect the environment, their collective efficacy is considered high. Collective efficacy encourages farmers to view collective action as an effective means of achieving the group’s goal—sustainable wetland protection and management. Farmers with high collective efficacy are more likely to engage in collective wetland management and conservation activities and to support and implement government policies within their communities (Thomas et al., 2012; Liu et al., 2020). Review of the literature (see Thomas et al., 2012; Bamberg et al., 2015; Valizadeh et al., 2022b; Valizadeh et al., 2022a; Meijers et al., 2023; Faghani et al., 2023; Johnson and Reimer, 2023; Gibson et al., 2024) indicates that there is a positive relationship between collective efficacy and intention. Therefore, we hypothesize that:

- Collective efficacy will positively affect the intention toward participation in management and protection of wetlands (H1).

### 2.2 The relationship between negative emotions and intention

So far, the role of emotions in the emergence of behavioral intentions toward collective actions has not been widely studied (Landmann and Rohmann, 2020). However, the influence of emotions in environmental communications and collective behaviors is increasingly becoming a research hotspot (Feldman and Hart, 2016; Nabi et al., 2018). Generally, negative emotions arise from feelings of perceived injustice in response to environmental issues, such as wetland destruction (Van Zomeren et al., 2019). Most existing literature on collective action (see Bamberg et al., 2015; Landmann and Rohmann, 2020; Meijers et al., 2023; Faghani et al., 2023; Waters et al., 2024) emphasizes group-based anger as a central negative emotion. In other words, these studies consider group-based anger a key factor in motivating participation in collective behaviors, such as wetland protection. Therefore, we hypothesized that:

- Negative emotions will negatively affect the intentions toward participation in management and protection of wetlands (H2).

### 2.3 The relationship between social identity and intention

The concept of social identity is shaped by the social context in which it develops (Delia and James, 2018). In general, social identity refers to a person’s perception of themselves as part of a group, forming an emotional connection with the group, and sharing similar characteristics, interests, and connections with other members of that group (Kural and Özbek, 2023; Zheng et al., 2023; de Hoog and Pat-El, 2024). Social identity theory highlights that individuals possess both personal and social identities. Personal identity includes traits such as abilities and interests, while social identity consists of significant group categories based on demographic factors (such as gender and race) or organizational memberships (such as environmental, educational, and social organizations) (Kural and Özbek, 2023). A review of research literature (see Binu Raj, 2021; Valizadeh et al., 2022a; Zeqiri et al., 2022; Faghani et al., 2023; Kural and Özbek, 2023) shows that social identity can have a direct effect and have a positive effect on environmental and community-oriented behaviors. Therefore, we hypothesized that:

- Social identity will positively affect intention toward participation in management and protection of wetlands (H3).

### 2.4 The relationship of collective efficacy and negative emotions with social identity

Social identity is not only a key predictor of collective action but also mediates the relationship between two other psychological factors—perceived negative emotions and collective efficacy—and the intention to participate in collective wetland protection and management activities. In this theory, social identity serves as both a direct and an indirect motivator for action. It directly motivates individuals by fostering a sense of belonging and shared responsibility within the group, and it indirectly influences participation by shaping negative emotions and collective efficacy at the group level, thereby activating the desire for collective action (Van Zomeren et al., 2008; Bamberg et al., 2015; Liu and Ravenscroft, 2017). According to Fritsche et al. (2018), in addition to their direct effects, collective efficacy and negative emotions can also indirectly influence participation in collective wetland protection and management through social identity. A review of research literature (see Setiawan et al., 2020; Keshavarzi et al., 2021; Valizadeh et al., 2022b; Pistoni et al., 2022) shows that collective efficacy and negative emotions directly affect social identity and indirectly strengthen the behavioral intention as well. Therefore, we hypothesized that:

- Collective efficacy will positively affect social identity about participation in collective management and protection of wetlands (H4).
- Negative emotions will negatively affect social identity about participation in collective management and protection of wetlands (H5).

In the field of environmental psychology, several theories have been proposed to explain human behavior, including the theory of planned behavior, reasoned action theory, norm activation theory, value-belief-norm theory, and protection motivation theory (Faghani et al., 2023; Valizadeh et al., 2022b). However, EMSICA may offer an alternative framework for understanding people’s behavior, as it emphasizes the role of group identity and individuals’ positions within a group (Bamberg et al., 2015; Keshavarzi et al., 2021). While many of the aforementioned behavioral theories focus on individual-level behavior (Bamberg et al., 2018; Valizadeh et al., 2022a), EMSICA considers behavior within a collective context. The EMSICA theory does not view people’s behavior in isolation but asserts that individuals’ relationships with the groups to which they belong can significantly influence their actions (Thomas et al., 2012). Many modern crises, such as wetland destruction, are complex, multi-stakeholder issues that cannot be addressed solely through individual-level approaches (Pistoni et al., 2022). Therefore, theories that focus on individual behavior may be insufficient for explaining such behavior (Fritsche et al., 2018). Using EMSICA in conjunction with other theories may provide a more comprehensive understanding of human behavior (Faghani et al., 2023).

## 3 Materials and methods

### 3.1 Research design

This research was a cross-sectional and quantitative study. Given that the results can be applied by various end-users, it is classified as applied research. Additionally, in terms of the degree of control over the variables, the study is non-experimental.

### 3.2 Study site

**Figure 2:**
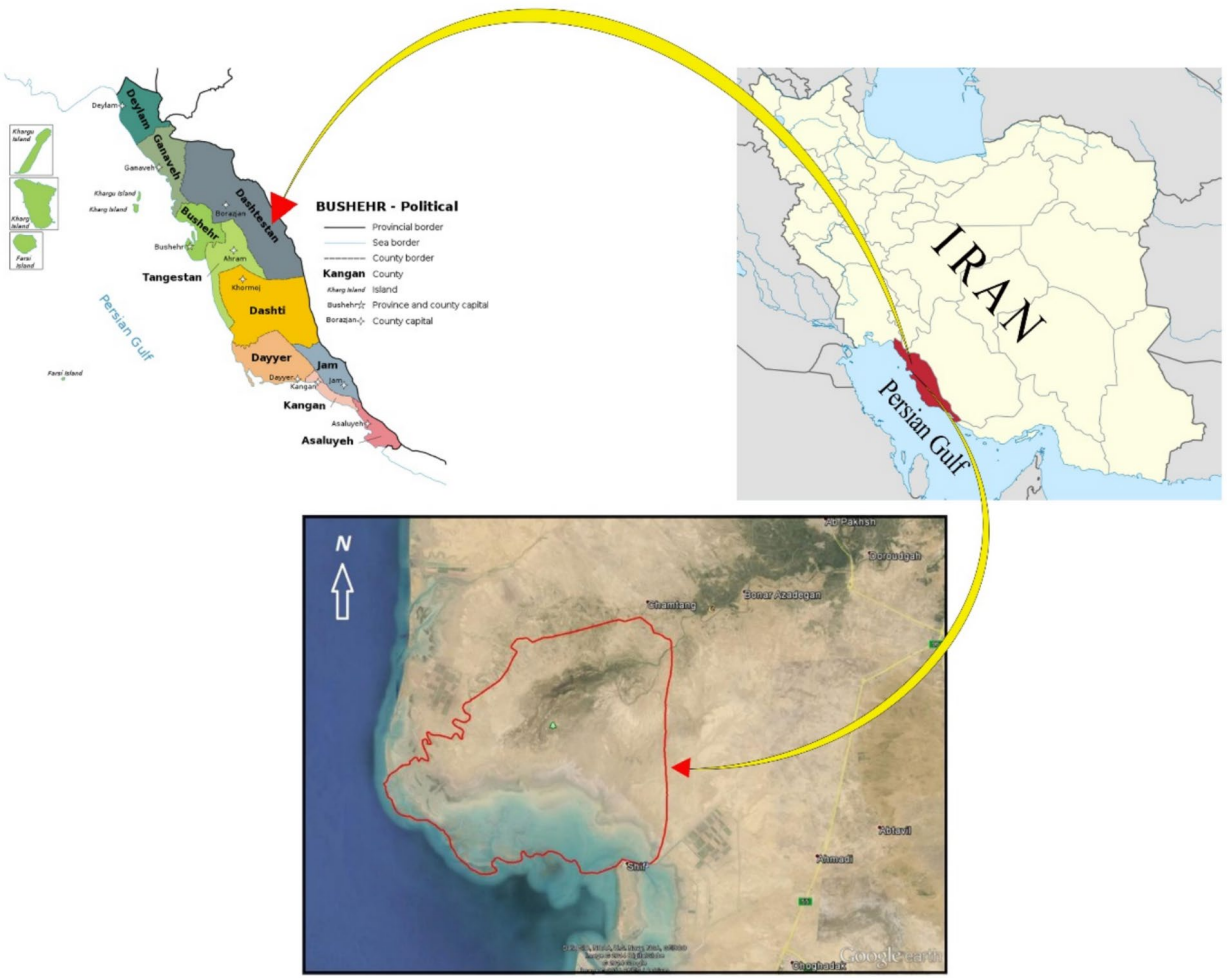
The map of the study area. This map includes data from: Google Data SIO, NOAA, U.S. Navy, NGA, GEBCO, landsat/Copernicus, Airbus, TerraMetrics, CNES/Airbus Imagery from the dates: 2015/14/12-2021/01/1.

Helleh Wetland emerged in 1963 due to the flooding of the Helleh River. Since then, it has been recognized as a wetland and a protected area in Bushehr province, Iran. Helleh Protected Area with an area of 46,783 ha is located in the geographical coordinates of “24′38 ° 50 to” 23′56 ° 50 east longitude and “24′03 ° 29 to” 50′16 ° 29 north latitude. Helleh Wetland is located 10 km northeast of Bushehr (Figure 2), with a depth of approximately 1.85 meters. The economy and livelihoods of the people around the wetland are primarily based on agriculture (including the cultivation of wheat, barley, and canola), animal husbandry (raising both light and heavy livestock), and horticulture (grove farming). Helleh River serves as a shared water source for both the agricultural sector and the wetland, meaning that the local economy, particularly agriculture, is closely tied to the wetland. Additionally, the reeds surrounding the wetland are used for livestock grazing. However, several factors have contributed to the degradation of the wetland in recent years. These include consecutive droughts, the conversion of wetland areas into agricultural lands, illegal hunting, the construction of the Rais Ali Delvari Dam on the Shapoor River, and the allocation of the wetland’s water resources for other purposes. As a result, the wetland has been severely disrupted and is now at risk of drying up. Other contributing factors include the pumping of water from the Hilleh River to supply water for shrimp ponds and the excessive pressure from farmers to meet the fodder needs of their livestock, which have further exacerbated the situation (Behroozi-Rad, 2009). In addition, factors such as plant diversity, the presence of the Helleh River, and the proximity to the shores of the Persian Gulf have made this wetland a rich habitat and grazing ground for wildlife. The vegetation in the wetland includes saline shrubs, forage plants, turmeric trees, walnuts, and various wetland plants. Aquatic plants in the wetland, along with the habitats of waterfowl and shorebirds, are considered endangered. The area is home to several animal species, including boars, jackals, wolves, foxes, and rabbits. Additionally, various bird species inhabit the protected area of the wetland, such as the Dragon, Lunar, Pigeon, Chirp, Cockatoo, Derna, Gray Goose, Flamingo, Pike, Gray Duck, Flat-tailed Duck, and Hubra (Behroozi-Rad, 2009). These features have prompted the Iranian government to develop programs aimed at protecting and ensuring the sustainable management of the wetland. As a result, increasing the participation of farmers in the surrounding areas has become a key component of these programs. Since no study has yet been conducted on the factors influencing farmers’ willingness to participate in the management and protection of this wetland, the Department of Environment of Iran has recognized this as a significant research gap and a priority.

### 3.3 Sampling method

The study population consisted of farmers living in the villages surrounding Helleh Wetland. Seven villages—Karehband, Mehrizi, Qaleh Sokhteh, Askari, Rostami, Farakeh, and Hamoud—are located around the wetland and are closely connected to it in terms of livelihood. In total, 1,876 people reside in these villages, with 536 of them being farmers. Given that the residents’ income is directly linked to the wetland, the study aimed to include all these areas. To determine the sample size, the Krejcie and Morgan table was used, which is a widely recognized method for calculating sample sizes in social studies. According to the table, 225 samples were estimated for a community of 536 individuals. After determining the sample size, a simple random sampling method was employed to select participants, due to the cultural, social, economic, and geographical similarities of the villages around Helleh Wetland. As a result, 225 farmers were randomly selected for the cross-sectional survey. When distributing the questionnaires and explaining the research objectives to participants, they were informed that participation was voluntary and there was no obligation to take part. They were also assured that not participating would have no negative consequences. Thus, respondents had complete freedom to choose whether or not to participate. Ultimately, all 225 individuals selected as part of the sample completed the questionnaire, resulting in a 100% participation rate.

### 3.4 Research instrument, data collection, and analysis

The tool used to collect information from farmers around the wetland was a close-ended, researcher-made questionnaire. A five-point Likert scale was employed to measure the items of the main variables. Participants were asked to respond to each question within a range of 1 (completely disagree) to 5 (completely agree). To ensure the validity and reliability of the questionnaire, various methods and indicators were applied. The face validity of the questionnaire was assessed through the input of experts and specialists. This diverse group included field experts and experienced faculty members with strong backgrounds in socio-environmental studies. Their valuable opinions and expertise were crucial in evaluating the questionnaire’s face validity. Additionally, a pilot study was conducted to assess the Cronbach’s alpha reliability index of the questionnaire. The variables, items developed to measure them, and their corresponding alpha coefficients are presented in Table 1. For this pilot study, a questionnaire with confirmed face and content validity was tested among a small sample of 30 farmers with characteristics similar to the target population. The results of the pilot study were used to calculate Cronbach’s alpha coefficients. In this process, Cronbach’s alpha coefficients were calculated for the variables measured using the Likert scale. The “if item deleted” option in SPSS software was activated to identify items that could significantly improve alpha coefficients. After making minor adjustments to the items or removing some, the research instrument was finalized for the main research phase. Finally, required data was collected and analyzed. It should also be mentioned that the authors used some brainstorming sessions in data analysis process to propose strategies for producing and reproducing social identity (see section 4). In this process, the authors have had several brainstorming sessions to propose these strategies (steps). This brainstorming session was conducted in three sessions with the participation of all authors. In presenting the main steps of producing and reproducing social identity, the first author played a key role due to his extensive experience in the social and psychological dimensions of the wetland communities. After summarizing the opinions of all participants in three brainstorming sessions, four steps (strategies) were presented for producing and reproducing social identity in the field of the wetland management and protection.

**Table 1:** The items used to measure the variables and reliability scores.

Var.	No.	Items	Source
IPCMP	**Intention toward participation in collective management and protection (IPCMP): (α = 0.81)**		Self-developed
	1	I would like to participate in management and protection of Helleh wetland	
	2	I would like to cooperate with the government, experts, and other stakeholders involved in the rehabilitation of Helleh Wetland	
	3	I would like to encourage other farmers to participate in the management and rehabilitation of Helleh Wetland	
	4	I would like to pay for the rehabilitation and protection of Helleh wetland	
	5	I want to learn the necessary skills for the protection and management of Helleh wetland	
PCEPMP	**Collective efficacy: (α = 0.77)**		Self-developed
	1	Through collective actions in management and protection of Helleh wetland, it is possible to effectively help reduce its problems	
	2	Collective measures significantly facilitate the efficacy of management and protection process of Helleh wetland	
NEPCMP	**Negative emotions: (α = 0.83)**		Self-developed
	1	I find it unlikely that all farmers and other stakeholders (government and private sector) will participate in management and protection of Helleh wetland	
	2	The lack of commitment of one of the stakeholders to the process of management and protection of Helleh wetland makes me nervous	
	3	Violation of any of the stakeholders from the rules and regulations set for management and protection of Helleh Wetland will cause me to violate the rules as well	
SIPCMP	**Social identity: (α = 0.73)**		Self-developed
	1	I will be happy to participate as a member of a group in management and protection of Helleh Wetland	
	2	Participating and playing a role in management and protection of Helleh Wetland is an important part of my self-image	
	3	I feel that I have strong ties to the people who participate in management and protection of Helleh Wetland	

Partial Least Squares Structural Equation Modeling (PLS-SEM), AMOS 26, SPSS 23, and Excel software were used to analyze the data and test the hypotheses. Kurtosis and skewness values were applied to assess the normality of the data collected from farmers. Additionally, standardized residual covariance was used to verify the normal distribution of residual covariance. Normality indices were estimated using AMOS_26_. In Structural Equation Modeling (SEM), both measurement and structural models were employed to test the relationships among the variables. The measurement and structural models for the study were executed using PLS-SEM. In this model, construct reliability and validity were assessed using Composite Reliability (CR) and Average Variance Extracted (AVE). Additionally, discriminant validity was evaluated through the Fornell-Larcker Criterion, the Heterotrait-Monotrait Ratio (HTMT), and Collinearity Statistics (VIF).

Smart-PLS is a statistical software widely used in the social sciences and other scientific disciplines to analyze complex relationships between variables. Its use was particularly beneficial in this study for several reasons. First, PLS enables researchers to simultaneously examine multiple relationships between predictor and response constructs. For example, in this study, Smart-PLS helped the researchers analyze both direct and indirect relationships between constructs through bootstrapping and mediation analysis. This capability allowed the researchers to explore interactions between different constructs. A second reason for using Smart-PLS was its ability to manage and reduce the effects of collinearity. Collinearity occurs when the predictor variables in a regression model are highly correlated with one another. Investigating this phenomenon is crucial in studies that analyze relationships between variables, as it can distort the results and interpretations. In fact, Smart-PLS manages collinearity more effectively than traditional regression methods by estimating latent variables that capture the common variance between correlated variables. This allows for better handling of multicollinearity. The third reason for using Smart-PLS in this study was its validation and predictive power. The software enables researchers to evaluate model fit and assess predictive power. Using Smart-PLS, researchers can examine how well the model fits the data, assess the importance of relationships between variables, and test the accuracy of the model’s predictions. The fourth reason for using Smart-PLS was its ability to aid in theory building. The software allows researchers to test and refine theoretical models, identify key relationships, and generate new hypotheses. Its flexibility in managing complex models and providing valuable insights into relationships between variables makes it a powerful tool for advancing social identity theories and other theoretical frameworks.

## 4 Results

The study findings were presented and thoroughly analyzed across several sub-sections, ensuring a comprehensive examination. These sub-sections covered important aspects such as the assessment of normality, the estimation of standardized residual covariance, and the evaluation of measurement and structural models. By addressing these key components, the study offered a detailed framework for reporting and discussing the results, facilitating a deeper understanding and interpretation of the research outcomes.

### 4.1 Assessment of normality

The results of the data normality evaluation in structural equation modeling are presented in Appendix 1. Kurtosis and skewness, which are two key indicators for assessing normality, should ideally fall between −3 and +3. Since all observed results for the EMSICA items fall within this range, it can be concluded that the data follow a normal distribution. Therefore, the results of the structural equation modeling can be analyzed with a relatively high degree of confidence.

### 4.2 Estimation of standardized covariances of residuals

The standardized covariance of residuals for the items measuring the variables is presented in Appendix 2. In the symmetric matrix shown in Appendix 2, each residual covariance for an item is divided by its standard error estimate. It is important to note that in sufficiently large samples, these standardized residual covariances follow a standard normal distribution if the model is statistically correct (Collier, 2020). Therefore, if the model is correct, most of the covariances should have an absolute value less than two. As shown in Appendix 2, most of the standardized residual covariances are below two. This suggests that the estimated model is correct and that the standardized residual covariance follows a standard normal distribution. Although the standard normal distribution of the residual covariance matrix has been confirmed in this study, four values in the matrix exceeded the acceptable cutoff value of two (highlighted in bold in Appendix 3). Despite this, we retained the items corresponding to these values in the analysis, as their deviation from the acceptable threshold was small.

### 4.3 Measurement models

In estimating and applying structural equation modeling, the internal and external relationships of variables and items were examined using measurement models (see Appendix 3). This study aimed to test the hypothetical relationships between the EMSICA variables and the items developed to measure them, using cross-sectional data collected from areas around Helleh Wetland.

#### 4.3.1 Evaluation of the construct reliability and validity

The findings from the measurement models indicated that all items showed an acceptable correlation with the EMSICA variables. This suggests that the items within each variable are strongly associated with the respective variable. In other words, the first-order measurement models confirmed that the number of latent variables and the items loading on them align with the expectations based on the EMSICA framework. Additionally, the construct reliability and validity of the EMSICA variables were assessed using Composite Reliability (CR) and Average Variance Extracted (AVE). The results showed that the observed values of the AVE index for the variables intention, perceived collective efficacy, and social identity are greater than the assumed acceptable value (0.5). It should be noted that at this stage, the AVE value for the construct negative emotions was less than the acceptable value of 0.5. Therefore, the item “Violation of any of the stakeholders from the rules and regulations set for management and protection of Helleh Wetland will cause me to violate the rules as well” which had a lower factor loading than the other items was removed from the analysis. Removing this item increased the AVE value of negative emotions. Also, the observed CR values were higher than the assumed values in the measurement models (0.7) (Appendix 3). Therefore, the construct reliability and convergent validity of the variables in the EMSICA was confirmed.

#### 4.3.2 Evaluation of the discriminant validity

The Fornell-Larcker Criterion was the first method used to test the discriminant validity of the constructs. According to the results, if a construct’s Fornell-Larcker criterion value is greater than its correlation with other variables, it indicates that the discriminant validity of the constructs has been satisfied (Appendix 4). The second important criterion used to test discriminant validity was the HTMT ratio. The HTMT ratio should be less than 1 for a construct to have acceptable discriminant validity. The results showed that all constructs had HTMT values below the cut-off of 1, meaning the HTMT-based discriminant validity was also fulfilled in this study (Appendix 5). VIF (Variance Inflation Factor) is another key criterion for examining discriminant validity in structural models. In this study, VIF was assessed for both the structural and measurement models. The results indicated that all VIF values for the inner model were below 2 (Appendix 6), meaning there was no significant variance inflation among the items of the measurement model. Furthermore, VIF values for the structural model were also below 2 for all constructs (Appendix 7), confirming that there was no significant variance inflation among the latent variables. Therefore, it can be concluded that the discriminant validity of the items and constructs used in this study was confirmed.

### 4.4 Structural model

**Figure 3:**
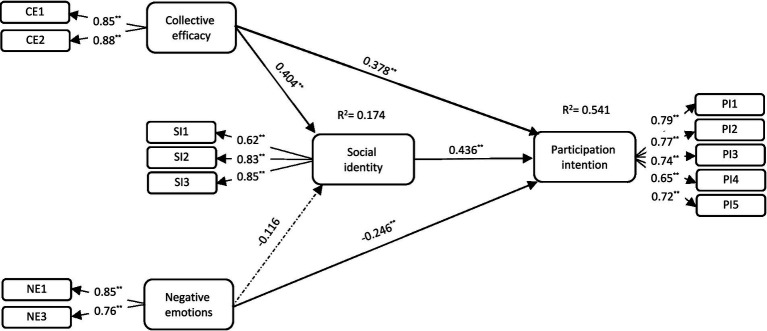
PLS-SEM of the research framework.

At this stage, the study framework was implemented using a structural model (Figure 3). This model was an indirect model because the SIPCMP mediates the relationship between NEPCMP and PCEPMP. The results of this section are presented in Table 2. The first part of the EMSICA mediation model showed that the relationship between PCEPMP and SIPCMP was significant (β = 0. 404; *p* > 0.01). However, the relationship of NEPCMP and SIPCMP was not statistically significant (β = −0. 116; n.s.). In other words, the hypothesis NEPCMP→IPCMP was rejected and the hypothesis PCEPMP→SIPCMP was confirmed. The second part of the structural model showed that the relationship of NEPCMP (β = −0. 246; *p* > 0.01), SIPCMP (β = 0. 436; *p* > 0.01), and PCEPMP (β = 0. 378; *p* > 0.01) with IPCMP are statistically significant. These results mean that the three hypotheses NEPCMP→IPCMP, SIPCMP→IPCMP, and PCEPMP→IPCMP were confirmed. The results of EMSICA structural analysis demonstrated that the predicting variables were able to predict 54.1 and 17.4% of the variance changes of IPCMP and SIPCMP, respectively. A comparison of the standardized path values of the predicting variables on the IPCMP reveals that SIPCMP and PCEPMP are the strongest predictors of both SIPCMP and IPCMP (Table 2; Figure 3).

**Table 2:** Estimated relationship among predicting variables and response variables (social identity and intention).

Dependent variable	Hypothesis	Direct relationship value		Indirect relationship value		Standard deviation	Total relationship value	*R* ^2^	Result
		*T*	Beta	*T*	Beta				
Social identity	NEPCMP → SIPCMP	1.691	−0.116	–	–	0.065	−0.116	0.174	Rejected
	PCEPMP → SIPCMP	6.882^**^	0.404			0.065	0.404		Accepted
Intention	SIPCMP → IPCMP	6.813^**^	0.436	–	–	0.056	0.436	0.541	Accepted
	PCEPMP → IPCMP	6.836^**^	0.378	–	–	0.059	0.554		Accepted
	NEPCMP → IPCMP	4.369^**^	−0.246	–	–	0.055	−0.296		Accepted
	PCEPMP → SIPCMP → IPCMP	–	–	4.511^**^	0.176	0.039	–		Accepted
	NEPCMP → SIPCMP → IPCMP	–	–	1.659	−0.050	0.030	–		Rejected

ns, not significant; **p < 0.01.

IPCMP, Intention toward participation in collective management and protection; PCEPMP, Collective efficacy about participation in management and protection; NEPCMP, Negative emotions about participation in collective management and protection; SIPCMP, Social identity about participation in collective management and protection.

Figure 3 also illustrates the significance or non-significance of the relationships between the latent variables, as well as the relationships of the indicators with the latent variables. In this figure, a *T*-value of 1.96 or greater indicates statistical significance. Upon examining the relationships between the latent variables and their indicators, all *T*-values were found to be greater than 1.96, meaning that the correlation values are considered significant. Further analysis of the relationships between the latent variables in the framework reveals that only the effect of negative emotions on social identity is less than the acceptable value, indicating that this relationship is not significant. Non-significant relationships or paths are represented by dashed lines in Figure 3, while significant relationships or paths are shown with solid lines and two asterisks above the path coefficients and factor loadings.

## 5 Discussion and international policy implications

The results indicated that collective efficacy has a positive and significant relationship with both intention and social identity. It also exhibits the greatest explanatory power in the model. Based on these findings, it can be concluded that improving the collective and participatory efficacy of farmers could address some issues related to the management and protection of wetlands. A similar result can be seen in the findings of researchers such as Thomas et al. (2012), Bamberg et al. (2015), Yazdanpanah et al. (2015), Maleksaeidi et al. (2015), Valizadeh et al. (2022b), Faghani et al. (2023), Meijers et al. (2023), Johnson and Reimer (2023), and Gibson et al. (2024). These researchers’ findings reveal that one of the major factors damaging natural resources, such as wetlands, is the lack of a proper understanding of the efficacy and effectiveness of collective measures in wetlands protection programs. In other words, enhancing the collective efficacy of farmers in managing and conserving wetlands can help sustain the region’s ecosystem and livelihood resources. Additionally, collective efficacy plays a crucial role in strengthening social identity. Therefore, it is recommended that the responsible organizations and institutions focus on strengthening collective efficacy in the management and protection of wetlands. Intervening organizations can adopt effective strategies to strengthen collective efficacy among farmers. To do so, they must establish structures that support collaborative processes for managing and protecting wetlands. This will help enhance literacy, knowledge, and skills—essential elements for achieving the desired collective efficacy within the agricultural community for wetlands management and protection.

Negative emotions had also a direct relationship with intention. Similar results can be seen among the results of Bamberg et al. (2015), Landmann and Rohmann (2020), Meijers et al. (2023), Faghani et al. (2023), and Waters et al. (2024). In many areas, negative emotions about collective action arise from past experiences with participating in such efforts. To address this, it is recommended that managers and implementers of wetland protection and management programs work to alleviate the negative memories farmers may have. The first step is to conduct a participatory appraisal of the wetland management and protection programs, where farmers can clearly understand the role and importance of their participation. In this process, farmers actively engage in the programs, which helps reduce negative emotions. This reduction in negative feelings will, in turn, improve their willingness to participate in the management and protection of wetlands.

The results showed that as social identity increases, intention also rises. This indicates that farmers’ willingness to participate in collective wetland management and protection activities is largely influenced by their social identity. These results align with the findings of Binu Raj (2021), Valizadeh et al. (2022a), Zeqiri et al. (2022), Faghani et al. (2023), and Kural and Özbek (2023). These studies suggest that social identity beliefs play a key role in reducing conflicts between farmers and other stakeholders in wetland management and protection programs. In other words, social identity helps bring individuals together under a shared identity based on society and common interests. The development of such an identity can encourage cooperation and empathy among members of farming communities. One effective way to strengthen social identity is through the formation of farmers’ organizations and unions. In these groups, each farmer has a specific and predefined role that aligns with the collective efforts in wetland management and protection, fostering a sense of identification. Furthermore, these organizations and unions empower farmers to more effectively influence decisions and major activities related to wetland management and protection. Managers and decision-makers involved in wetlands management and protection can implement various strategies to enhance and even cultivate social identity (to see the process and methodology of these steps, refer to section 3.4).

In many cases, farmers lack a social identity for collective action in wetland management. Therefore, wetland managers and policymakers should first shift from top-down decision-making systems to bottom-up approaches. This transition is the first practical step in fostering and institutionalizing social identity. The second step involves creating and reinforcing social identity in alignment with farmers’ agricultural priorities and goals. In other words, social identity should be reproduced in a way that minimizes costs—financial, intellectual, and temporal—especially in the early stages of social change. Achieving this step can, in the short term, reduce conflicts of interest between farmers and other stakeholders involved in wetland management and protection. Over time, this process can cultivate a sense of “we thinking” among stakeholders. In the third step of reproducing and institutionalizing social identity, the key leaders of collective management and protection programs should aim to reduce their direct involvement in decision-making and the implementation of practical measures. This approach helps local communities feel that they are not just passive observers. By participating in the decisions and actions of wetland management programs, they begin to recognize that they are key players in the program, which contributes to the development of their identity within these initiatives. Currently, farmers serve as active participants in the collective management and protection of wetlands, while external actors mainly act as advisors and supervisors. This collaborative model values the expertise and experience of local farmers, involving them directly in decision-making processes. As the initiative moves into the fourth stage, it is crucial to establish a well-defined exit strategy that ensures the full transfer of authority to farmers and local communities. This exit strategy requires careful planning and consideration. Its goal is to empower farmers and the local community to independently take on full responsibility for wetland management and protection. This transition of authority will promote long-term sustainability and ensure that collective efforts continue effectively even without external intervention. By creating a clear roadmap for this shift, which includes capacity-building, knowledge transfer, and institutional support, the exit strategy sets the foundation for a self-governing and self-sustaining collective management system led by farmers and local communities.

The effect of negative emotions on social identity was not significant. One possible reason for the lack of a significant relationship between these two factors is that the communities around the wetland view wetland management and protection as a matter of personal opinion. In other words, they do not perceive the participation of all stakeholders in wetland management and protection as a necessity for everyone. As a result, negative emotions regarding the non-participation of stakeholders in these efforts are reduced. This factor also weakens the ability of negative emotions to significantly impact social identity. Additionally, the non-significance of this effect could be attributed to the nature of the items used to measure negative emotions. These items may not effectively capture the full scope of negative emotions experienced by the participants.

Several important factors limited the present study. First, the encapsulation model of social identity in collective action was used to conceptualize farmers’ intention to manage and protect the wetlands. However, this model offers a simplified framework for studying collective behaviors and their predictors. As a result, the model is open to further development by incorporating additional variables. For instance, future studies could expand the model by including variables such as socialization, participatory efficacy, and regulatory focus. The second limitation concerns the subjectivity in analyzing intention. Specifically, the study focused on subjective reports of intention, without examining the actual behavior of farmers. Future researchers could replace the variable of intention toward wetland management and protection with actual behavior within the framework. The third limitation of this study is that it only examines the relationship between a few psychological variables and intention. Therefore, future research could explore how economic, cultural, religious, and social variables influence intention. Another limitation is the use of researcher-made items to measure the latent variables in this study. While all variables showed high internal consistency and sufficient face validity, the non-significant effect of negative emotions on social identity might be due to the fact that the items for negative emotions did not fully capture the latent variables. Future researchers are encouraged to reexamine the effect of negative emotions on social identity by revising and validating the items used. Finally, one of the most significant limitations of this study is the lack of attention to the various identities of farmers, such as generation, ethnicity, race, religion, gender, sexual orientation, profession, and socio-economic status. It is recommended that future studies expand this framework to consider these different identities. Including such factors could provide more comprehensive and realistic insights into the topic.

## 6 Conclusion

The main aim of this study was to examine the intention to participate in the collective management and protection of wetlands. It was analyzed through the framework of the encapsulation model of social identity in collective action. The research resulted in four important conclusions. These conclusions can be valuable for managers, policy-makers, practitioners, and researchers involved in wetland management and protection projects. First, social identity models, such as the encapsulation model of social identity in collective action, can play a crucial role in supporting the collective management and protection of wetlands. In agricultural and local communities, these models help foster a “we” mentality in the context of wetlands management. This mindset encourages collaboration between farmers, local communities, and other stakeholders. It helps align their goals and reduces conflicts over the sustainable use of wetlands. Second, for the first time, four key steps were proposed to promote the reproduction of social identity in collective wetlands management and protection within agricultural and local communities. These steps are as follows: (1) Shifting from top-down to bottom-up decision-making systems to create a foundation for collective action, (2) Aligning the production and reproduction of social identity with the agricultural priorities and goals of farmers, (3) Gradually reducing the involvement of key stakeholders in decision-making and the implementation of practical measures, and (4) Developing an appropriate exit strategy to transfer full authority to farmers and the local community. These four stages of social identity reproduction in wetlands management are a significant innovation in this study. They can be applied in practice to manage and protect wetlands and other natural resources effectively. Third, collective efficacy plays a crucial role in any behavioral change program, whether for actual or future participation. It is a key factor in strengthening the intention to engage in collective management and protection. This can occur directly or indirectly, through the influence of social identity. Tangible changes in both intention and social identity can only be expected when participants understand the long-term and short-term effectiveness of collective wetland management. Fourth, incomplete or incorrect cooperation between stakeholders (both public and private) and farmers in wetland management and protection can create negative emotions. These emotions can significantly reduce farmers’ desire to participate and harm their social identity.

## Data Availability

The raw data supporting the conclusions of this article will be made available by the authors, without undue reservation.
