# Supplementary material for: From collective efficacy and negative emotions toward management and conservation of wetlands: the mediating role of social identity
Source: Front Psychol. 2025 Mar 14;16:1362750. doi: 10.3389/fpsyg.2025.1362750 (PMC11952117; doi:10.3389/fpsyg.2025.1362750)
Supplement: Supplementary file 1 [file Table_1.docx]

**Appendix 1**

| **Loading factors or correlation of items with the latent variables and validity and reliability analysis results** | | | | | | |
| --- | --- | --- | --- | --- | --- | --- |
| **Items** | min | max | skew | c.r. | kurtosis | c.r. |
| IPCMP1 | 2 | 5 | -0.061 | -0.367 | 0.682 | 2.064 |
| IPCMP2 | 2 | 5 | -0.235 | -1.424 | 0.125 | 0.379 |
| IPCMP3 | 2 | 5 | -0.810 | -4.902 | 1.387 | 4.201 |
| IPCMP4 | 1 | 5 | -0.677 | -4.100 | -0.195 | -0.589 |
| IPCMP5 | 1 | 5 | -1.013 | -6.136 | 2.230 | 6.752 |
| SIPCMP1 | 1 | 5 | -1.314 | -7.958 | 1.494 | 4.525 |
| SIPCMP2 | 1 | 5 | -0.874 | -5.294 | 0.758 | 2.294 |
| SIPCMP3 | 2 | 5 | -0.856 | -5.185 | 1.031 | 3.120 |
| NEPCMP1 | 1 | 5 | 0.904 | 5.475 | 0.688 | 2.082 |
| NEPCMP2 | 1 | 5 | 0.543 | 3.286 | -0.199 | -.602 |
| NEPCMP3 | 1 | 5 | 0.948 | 5.739 | 1.016 | 3.078 |
| PCEPMP1 | 2 | 5 | -0.423 | -2.563 | -0.159 | -0.480 |
| PCEPMP2 | 2 | 5 | -0.770 | -4.662 | 1.058 | 3.205 |
| Multivariate |  |  |  |  | 27.878 | 10.469 |
| * Fixed item  Abbreviations: IPCMP: Intention towards participation in collective management and protection, PCEPMP: Collective efficacy about participation in management and protection, NEPCMP: Negative emotions about participation in collective management and protection, SIPCMP: Social identity about participation in collective management and protection | | | | | | |

**Appendix 2**

| **Standardized covariances of residuals for the items** | | | | | | | | | | | |  |  |
| --- | --- | --- | --- | --- | --- | --- | --- | --- | --- | --- | --- | --- | --- |
| Items | IPCMP1 | IPCMP2 | IPCMP3 | IPCMP4 | IPCMP5 | SIPCMP1 | SIPCMP2 | SIPCMP3 | NEPCMP1 | NEPCMP2 | NEPCMP3 | PCEPMP1 | PCEPMP2 |
| IPCMP1 | 0.135 |  |  |  |  |  |  |  |  |  |  |  |  |
| IPCMP2 | 0.271 | 0.06 |  |  |  |  |  |  |  |  |  |  |  |
| IPCMP3 | 0.157 | -0.39 | -0.20 |  |  |  |  |  |  |  |  |  |  |
| IPCMP4 | -0.34 | -0.42 | 0.399 | -0.70 |  |  |  |  |  |  |  |  |  |
| IPCMP5 | 0.039 | -0.50 | -0.49 | 0.417 | -0.10 |  |  |  |  |  |  |  |  |
| SIPCMP1 | -0.73 | -0.35 | -0.57 | 0.370 | -0.06 | 0.066 |  |  |  |  |  |  |  |
| SIPCMP2 | -1.22 | -0.65 | 0.438 | -0.39 | 0.007 | 0.839 | -0.05 |  |  |  |  |  |  |
| SIPCMP3 | -0.93 | -0.29 | 0.600 | -0.17 | 0.143 | -0.10 | 0.10 | -0.05 |  |  |  |  |  |
| NEPCMP1 | -0.53 | -0.91 | -0.78 | 0.773 | 0.563 | 0.300 | 0.075 | 0.075 | 0.060 |  |  |  |  |
| NEPCMP2 | -1.22 | -0.32 | -0.12 | **2.428** | 0.887 | 0.147 | 1.680 | 1.168 | 0.124 | 0.011 |  |  |  |
| NEPCMP3 | **2.35** | **2.13** | 0.831 | **2.040** | 0.237 | -1.93 | 0.492 | 0.492 | -0.37 | 0.644 | 0.052 |  |  |
| PCEPMP1 | 0.338 | 0.454 | -0.68 | 1.418 | 0.925 | 0.300 | 0.059 | 0.059 | -0.12 | -0.55 | 1.398 | -0.06 |  |
| PCEPMP2 | 0.393 | 0.831 | -1.11 | -0.14 | 0.088 | -0.15 | 0.299 | 0.299 | 0.122 | -0.46 | 1.383 | -0.19 | 0.000 |
| * Fixed item  Abbreviations: IPCMP: Intention towards participation in collective management and protection, PCEPMP: Collective efficacy about participation in management and protection, NEPCMP: Negative emotions about participation in collective management and protection, SIPCMP: Social identity about participation in collective management and protection | | | | | | | | | | | | | |

**Appendix 3**

| **Loading factors or correlation of items with the latent variables and validity and reliability analysis results** | | | | |
| --- | --- | --- | --- | --- |
| **Items** | **IPCMP** | **SIPCMP** | **NEPCMP** | **PCEPMP1** |
| IPCMP1 | 0.79 |  |  |  |
| IPCMP2 | 0.77 |  |  |  |
| IPCMP3 | 0.74 |  |  |  |
| IPCMP4 | 0.65 |  |  |  |
| IPCMP5 | 0.71 |  |  |  |
| SIPCMP1 |  | 0.62 |  |  |
| SIPCMP2 |  | 0.83 |  |  |
| SIPCMP3 |  | 0.85 |  |  |
| NEPCMP1 |  |  | 0.85 |  |
| NEPCMP2 |  |  | 0.76 |  |
| PCEPMP1 |  |  |  | 0.85 |
| PCEPMP2 |  |  |  | 0.88 |
| CR | 0.85 | 0.81 | 0.79 | 0.86 |
| AVE | 0.54 | 0.60 | 0.65 | 0.75 |
| Abbreviations: IPCMP: Intention towards participation in collective management and protection, PCEPMP: Collective efficacy about participation in management and protection, NEPCMP: Negative emotions about participation in collective management and protection, SIPCMP: Social identity about participation in collective management and protection | | | | |
|  | | | | |

**Appendix 4**

| **Results of Fornell-Larcker Criterion values to evaluate the discriminant validity** | | | | |
| --- | --- | --- | --- | --- |
|  | **IPCMP** | **PCEPMP** | **NEPCMP** | **SIPCMP** |
| **IPCMP** | 0.738 |  |  |  |
| **PCEPMP** | 0.545 | 0.869 |  |  |
| **NEPCMP** | -0.279 | 0.031 | 0.808 |  |
| **SIPCMP** | 0.612 | 0.400 | -0.103 | 0.775 |
| Abbreviations: IPCMP: Intention towards participation in collective management and protection, PCEPMP: Collective efficacy about participation in management and protection, NEPCMP: Negative emotions about participation in collective management and protection, SIPCMP: Social identity about participation in collective management and protection | | | | |

**Appendix 5**

| **Results of HTMT values to evaluate the discriminant validity** | | | | |
| --- | --- | --- | --- | --- |
|  | **IPCMP** | **PCEPMP** | **NEPCMP** | **SIPCMP** |
| **IPCMP** |  |  |  |  |
| **PCEPMP** | 0.736 |  |  |  |
| **NEPCMP** | 0.480 | 0.054 |  |  |
| **SIPCMP** | 0.824 | 0.578 | 0.182 |  |
| Abbreviations: IPCMP: Intention towards participation in collective management and protection, PCEPMP: Collective efficacy about participation in management and protection, NEPCMP: Negative emotions about participation in collective management and protection, SIPCMP: Social identity about participation in collective management and protection | | | | |

**Appendix 6**

| **The results of VIF values for the outer model** | |
| --- | --- |
| Item | VIF |
| IPCMP1 | 1.915 |
| IPCMP2 | 1.828 |
| IPCMP3 | 1.513 |
| IPCMP4 | 1.343 |
| IPCMP5 | 1.409 |
| NEPCMP1 | 1.105 |
| NEPCMP2 | 1.105 |
| PCEPMP1 | 1.178 |
| PCEPMP2 | 1.461 |
| SIPCMP1 | 1.357 |
| SIPCMP2 | 1.357 |
| SIPCMP3 | 1.426 |
| Abbreviations: IPCMP: Intention towards participation in collective management and protection, PCEPMP: Collective efficacy about participation in management and protection, NEPCMP: Negative emotions about participation in collective management and protection, SIPCMP: Social identity about participation in collective management and protection | |

**Appendix 7**

| **Table 8. The results of VIF values for the inner model** | | | | |
| --- | --- | --- | --- | --- |
|  | **IPCMP** | **PCEPMP** | **NEPCMP** | **SIPCMP** |
| **IPCMP** |  |  |  |  |
| **PCEPMP** | 1.198 |  |  | 1.001 |
| **NEPCMP** | 1.017 |  |  | 1.001 |
| **SIPCMP** | 1.210 |  |  |  |
| Abbreviations: IPCMP: Intention towards participation in collective management and protection, PCEPMP: Collective efficacy about participation in management and protection, NEPCMP: Negative emotions about participation in collective management and protection, SIPCMP: Social identity about participation in collective management and protection | | | | |
